# Identification of Key Drug Targets and Molecular Mechanisms of Curcumae Rhizoma Acting on HBV-Related HCC: Weighted Correlation Network and Network Pharmacological Analyses

**DOI:** 10.1155/2022/5399766

**Published:** 2022-03-27

**Authors:** Mengyuan Zhao, Yun Fu, Lili Liu, Yong Hou, Mei Shi, Hao Zhou, Guoliang Zhang

**Affiliations:** ^1^Anhui University of Chinese Medicine, Hefei, China; ^2^Department of Infectious Disease, The First Affiliated Hospital of Anhui University of Chinese Medicine, Hefei, China

## Abstract

**Background:**

Hepatitis B virus (HBV)-related hepatocellular carcinoma (HCC) has poor prognosis and high mortality rate. Curcumae Rhizoma, a classic Chinese medicinal herb, is often used to treat tumors.

**Methods:**

Active ingredients of Curcumae Rhizoma were extracted from the Traditional Chinese Medicine Database and Analysis Platform (TCMSP) database, and potential targets were predicted by the TCMSP database and Swiss Target Prediction database. The key drug targets were filtered by intersecting predicted targets, DEGs, and genes in important modules from WGCNA. Besides, the key drug targets were used to construct a network of “herb-active ingredient-target-disease” interactions and subjected to enrichment analysis and protein-protein interaction (PPI) analysis. The hub targets based on PPI analysis was evaluated by the KMplotter database.

**Results:**

Three active ingredients of Curcumae Rhizoma were collected with OB ≥ 30% and DL ≥ 0.18, including hederagenin, wenjine, and bisdemethoxycurcumin. The key drug targets were mainly enriched in cell cycle checkpoint, DNA integrity checkpoint, and peptidyl-serine modification. Besides, Curcumae Rhizoma treatment of HBV-related HCC mainly involved the p53 signaling pathway and arachidonic acid metabolism. Finally, ESR1 and PTGS2 were identified as hub targets from PPI analysis. ESR1 was found to be correlated with survival in liver cancer patients with hepatitis.

**Conclusion:**

Based on WGCNA and network pharmacological analysis, our results illustrated that Curcumae Rhizoma might work through regulating multitargets and multipathways in HBV-related HCC.

## 1. Introduction

Hepatocellular carcinoma (HCC) is currently recognized as one of the most hard-to-treat malignancies, with the incidence increasing significantly over the last century [1–3]. According to the 2018 global tumor statistics, HCC is the 4th leading cause of cancer death at 8.2% [4]. It was reported by the National Cancer Center in 2019 that there were 370000 new cases of HCC in 2015, making it the 4th most common malignant tumor and the 2nd leading cause of tumor death in China, seriously threatening human life and health [5, 6]. About 80% of HCC in China are caused by hepatitis B virus (HBV) infection, making HBV-related HCC one of the major public health problems in China [6]. However, its pathogenesis has not yet been fully understood.

In the traditional treatment of HBV-related HCC, chronic HBV infection and liver malignancy are seen as two causally related but relatively independent aspects, and therefore, their treatment is divided into long-term aggressive antiviral therapy for HBV and interventional, targeted, and surgical treatments for HCC [7, 8]. Existing Western medicine treatment programs are generally combinatorial rather than comprehensive. Traditional Chinese medicine (TCM) treatment, due to its holistic view of the disease, can play a comprehensive role in controlling disease progression in the context of chronic HBV infection, including blocking precancerous changes, compensating for the limitations of Western medicine alone, prolonging survival, and improving quality of life [9, 10]. However, due to the high complexity of TCM mechanisms, the exploration of drug targets and the screening of active ingredients is an important challenge at present.

Curcumae Rhizoma, bitter and acrid, could invigorate blood circulation, dispel blood stasis, regulate Qi, alleviate pain, dissolve accumulations, and alleviate pain [11, 12]. Curcumae Rhizoma has been reported to have anticancer activity against a variety of cancers, including breast cancer [13], gastric cancer [14], and colorectal cancer [15]. Curcumae Rhizoma or some of its active ingredients have been reported to have a better inhibitory effect on liver fibrosis and HCC [16, 17].

In this study, we obtained the GSE121248 dataset by searching datasets containing HCC tissues and paracancerous tissues with HBV infection in the GEO database. By bioinformatics techniques, we identified the significant modules and differentially expressed genes (DEGs) of the GSE121248 dataset. Using various databases and bioinformatics algorithms, we screened out active components of Curcumae Rhizoma, targets of HBV-related HCC, and key drug targets of Curcumae Rhizoma for the treatment of HBV-related HCC. The characteristics of these key targets were preliminary revealed. In summary, this study provides new targets and ideas for the treatment of HBV-related HCC with Curcumae Rhizoma.

## 2. Methods

### 2.1. Curcumae Rhizoma Active Ingredient Screening and Target Prediction

TCMSP is a traditional Chinese medicine database and analysis platform that could analyze the relationship among drugs, targets, and diseases, revealing the nature and potential mechanisms of TCM [18]. Curcumae Rhizoma was searched in the TCMSP database, and the chemical components were screened with oral bioavailability (OB) ≥ 30% and drug likeness (DL) ≥ 0.18%. The potential drug targets were also searched in the TCMSP database and Swiss Target Prediction database [19]. Then, the potential drug targets from the TCMSP database were imported into the UniProtKB database [20] for target gene name correction and elimination of nonhuman targets. The potential drug targets from the Swiss Target Prediction database were screened with probability > 0.

### 2.2. GSE121248 Dataset Collection

Gene Expression Omnibus (GEO) database [21] was utilized to search the public dataset associated with HBV-related HCC, and the GSE121248 dataset (https://www.ncbi.nlm.nih.gov/geo/query/acc.cgi?acc=GSE121248) provided by Wang et al. [22] was downloaded via R (version 3.6.3) package of GEOquery 2.54.1 [23]. Tissues from chronic hepatitis B induced HCC and their adjacent normal tissues were isolated, and total RNA was extracted for Affymetrix gene microarray analysis.

### 2.3. WGCNA

WGCNA is a systems biology approach to describe gene association patterns among different samples, to figure out highly synergistic gene sets, and to identify candidate biomarkers or therapeutic targets based on the endogeneity of the gene set and the association between the gene set and the phenotype [24]. The analysis methods were previously described [25]. WGCNA was performed using top 5000 genes with maximum mean absolute deviation, a power *β* of 6, a minimal module size of 30, a deep split of 3, and an unsigned type of topological overlap matrix (TOM). Finally, 13 modules were obtained, and Spearman correlations between modules and clinical features were analyzed.

### 2.4. Differential Expression Analysis

The expression matrix of the GSE121248 dataset was filtered by transforming repeated probe signals as the median value. The limma 3.42.2 package was then used for the differential expression analysis between tumor sample from hepatocellular carcinoma patient and adjacent normal sample from hepatocellular carcinoma patient. Besides, DEGs were screened with |logFC| ≥ 1 and *P* < 0.05 and visualized as a volcano plot. Top 50 DEGs were visualized as heatmaps with the clustering method of Euclidean distances using the ComplexHeatmap 2.2.0 package [26].

### 2.5. Construction of the “Herb-Active Ingredient-Target-Disease” Interaction Network

Key drug targets were screened by intersecting predicted targets, DEGs, and genes in important modules from WGCNA. Then, the interactions among herb, active ingredients, and targets were imported into the Cytoscape software (version 3.8.3) [27] for network construction.

### 2.6. Enrichment and PPI Analysis of Key Drug Targets

Key drug targets were subjected to Gene Ontology (GO) and Kyoto Encyclopedia of Genes and Genomes (KEGG) enrichment analysis using the clusterProfiler 3.14.3 package [28], and the top 10 entries with highest generation and *P* < 0.05 were visualized as bubble plots and chord diagrams.

Key drug targets were uploaded to the STRING database (version 11.0) [29], and interactions with a score above 0.4 were considered significant. Then, the interactions were downloaded and visualized using Cytoscape software (version 3.8.3) [27]. The common genes in top 5 genes with highest degree and bottleneck score were detected as hub targets by cytoHubba plugin [30].

### 2.7. Prognostic Analysis of Hub Targets

The hub targets were entered into the KMplotter database [31] and analyzed for prognostic value based on the hepatitis virus yes subgroup and all groups. The web was established to perform univariate and multivariate survival analyses using any custom-generated data.

### 2.8. Active Ingredient Screening and Target Prediction

**Figure 1:**
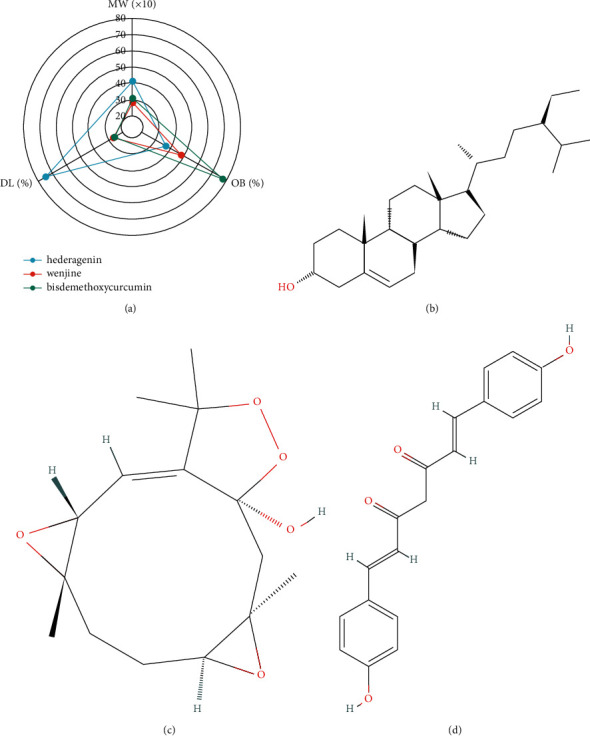
Curcumae Rhizoma active ingredients. (a) The characteristics of Curcumae Rhizoma active ingredients. (b) Hederagenin molecule structure. (c) Wenjine molecule structure. (d) Bisdemethoxycurcumin molecule structure.

The active ingredients of Curcumae Rhizoma, including hederagenin, wenjine, and bisdemethoxycurcumin, were obtained by searching the TCMSP database and screening with OB and DL parameters (Figure 1(a), Table 1). Their molecule structure was downloaded (Figures 1(b)–1(d)). Then, 22 potential targets of hederagenin were extracted from the TCMSP database. 11 potential targets of wenjine and 68 potential targets of bisdemethoxycurcumin were predicted from the Swiss Target Prediction database.

## 3. Results

### 3.1. HBV-Related HCC Target Screening

**Figure 2:**
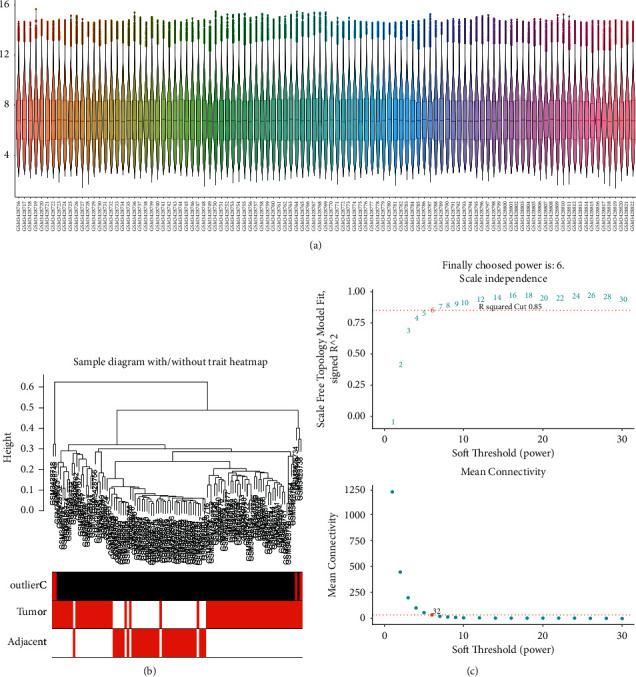
Expression profile preprocess and soft power determination. (a) The expression profile distribution of all liver samples. (b) Hierarchical clustering excluded outlier samples. (c) The soft-threshold power determined based on a scale-free *R*^2^ of 0.85.

**Figure 3:**
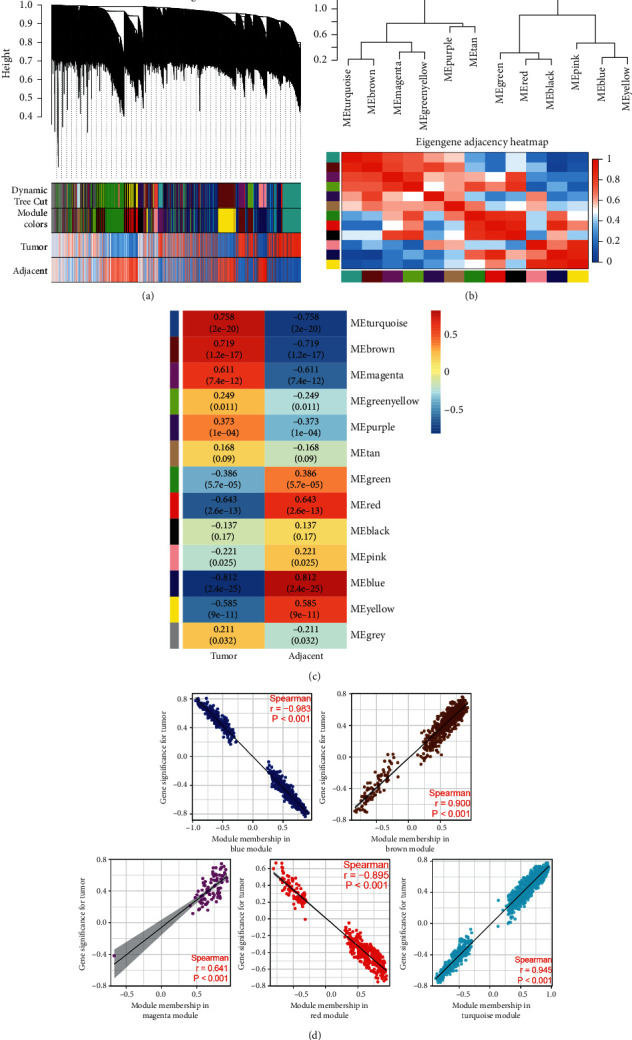
Coexpression module identification. (a) Dynamic tree cut. (b) Module correlation. (c) WGCNA module trait correlation plot with negative correlation plotted as blue color and positive correlation plotted as red color. (d) Correlation of module membership and gene significance in blue, brown, magenta, red, and turquoise modules.

To identify HBV-related HCC targets, we downloaded the GSE121248 dataset from the GEO database. By WGCNA analysis, a power *β* of 6 was detected and 13 modules were obtained (Figures 2, 3(a)–3(c)). Among these modules, blue, brown, magenta, red, and turquoise modules were screened as important modules since they were most significantly correlated with tumor (Figure 3(d)).

**Figure 4:**
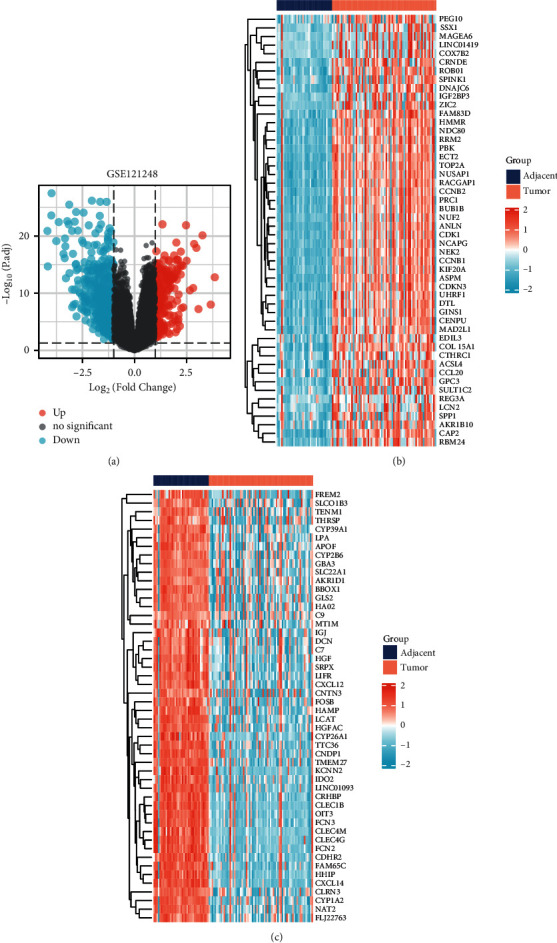
DEGs screening on the GSE121248 dataset. (a) Volcano plot with threshold of |logFC|≥1 and P adjust<0.05. (b) Top 50 upregulated genes. (c) Top 50 downregulated genes.

Then, expression profile of the GSE121248 dataset was subjected to differential expression analysis (Figure 4(a)). The top 50 upregulated (Figure 4(b)) and downregulated (Figure 4(c)) genes were visualized.

### 3.2. Key Drug Targets and Their Features

**Figure 5:**
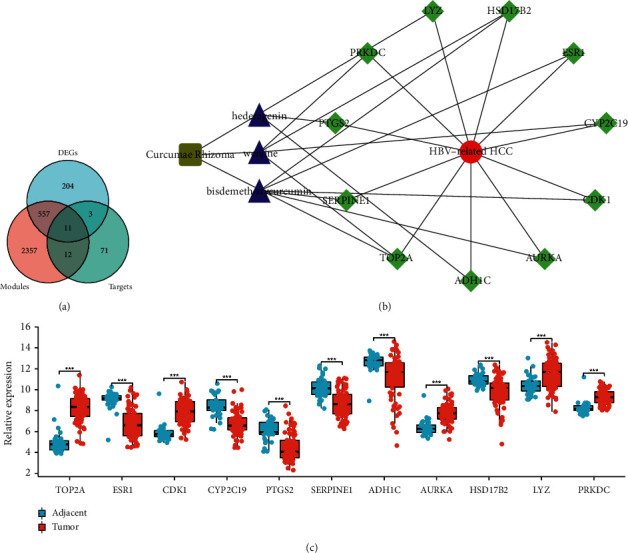
Key drug targets screening and network construction. (a) Key drug targets screening. (b) Key drug target expression in the GSE121248 dataset. (c) A network of “herb-active ingredient-target-disease” interactions.

To identify key drug targets of Curcumae Rhizoma in treating HBV-related HCC, we collected the overlapped genes in DEGs, genes in important modules, and predicted targets (Figure 5(a)). These genes were screened as key drug targets, including TOP2A, ESR1, CDK1, CYP2C19, PTGS2, SERPINE1, ADH1C, AURKA, HSD17B2, LYZ, and PRKDC (Table 2). Moreover, the expression levels of key drug targets were analyzed (Figure 5(b)). Then, a network of “herb-active ingredient-target-disease” interactions was constructed (Figure 5(c)).

**Figure 6:**
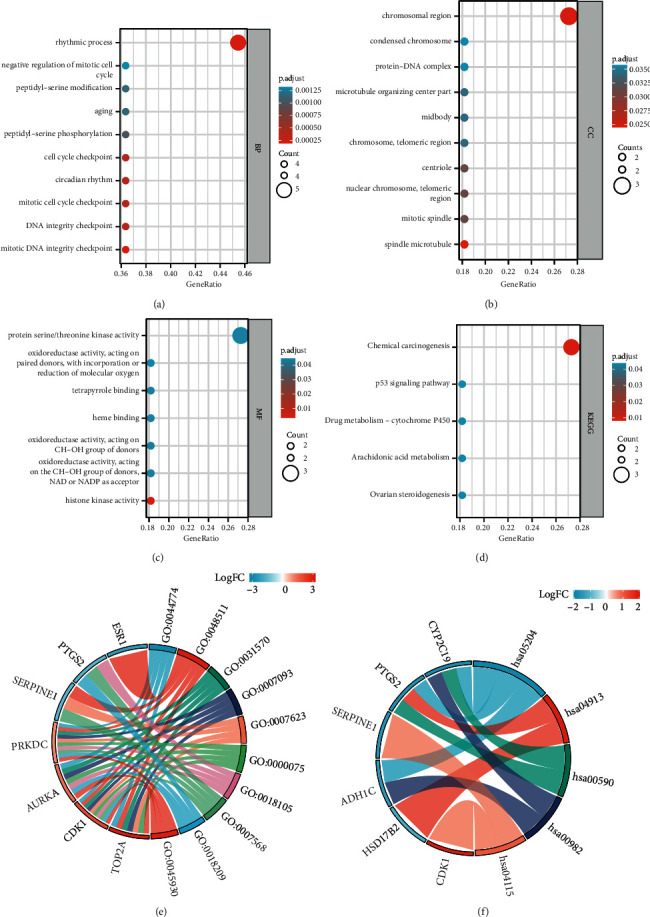
Enrichment analysis of key drug targets. (a) A bubble plot showing GO-BP analysis. (b) A bubble plot showing GO-CC analysis. (c) A bubble plot showing GO-MF analysis. (d) A bubble plot showing KEGG analysis. (e) A chord diagram showing GO-BP analysis. (f) A chord diagram showing KEGG analysis.

To characterize the key drug targets, we performed GO and KEGG enrichment analyses (Table 3). The results depicted that the key drug targets mainly involved in the rhythmic process, mitotic DNA integrity checkpoint, DNA integrity checkpoint, mitotic cell cycle checkpoint, circadian rhythm, cell cycle checkpoint, peptidyl-serine phosphorylation, aging, peptidyl-serine modification, and negative regulation of mitotic cell cycle (Figure 6(a)). Besides, some cellular component (CC) terms were also enriched, such as chromosomal region, spindle microtubule, mitotic spindle, nuclear chromosome, telomeric region, centriole, chromosome, telomeric region, midbody, microtubule organizing center part, protein-DNA complex, and condensed chromosome (Figure 6(b)). In terms of molecular function (MF), key drug targets participated in protein serine/threonine kinase activity, histone kinase activity, oxidoreductase activity, acting on the CH-OH group of donors, NAD or NADP as acceptor, oxidoreductase activity, acting on CH-OH group of donors, heme binding, tetrapyrrole binding, and oxidoreductase activity, acting on paired donors, with incorporation or reduction of molecular oxygen (Figure 6(c)). Moreover, the involved pathways included chemical carcinogenesis, ovarian steroidogenesis, arachidonic acid metabolism, drug metabolism-cytochrome P450, and p53 signaling pathway (Figure 6(d)). Combining logFC, the GO-biological process (BP) and KEGG enrichment results are shown in Figures 6(e) and 6(f).

**Figure 7:**
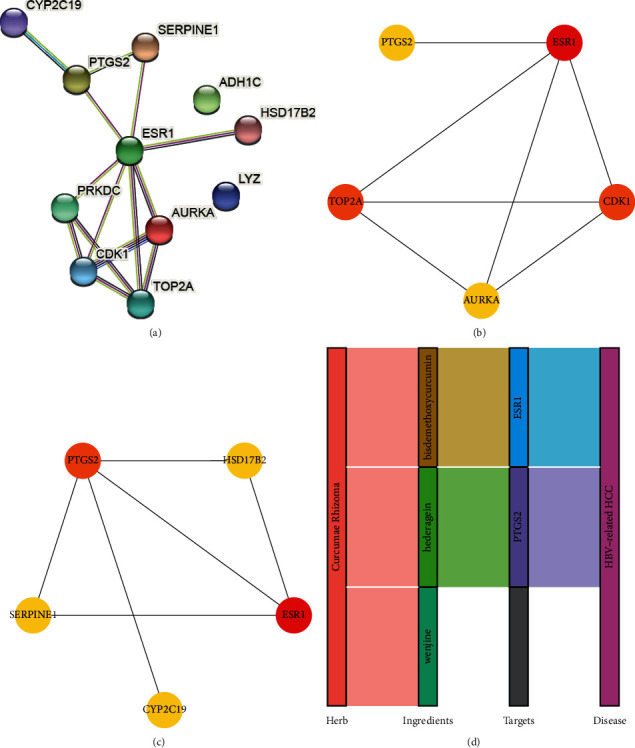
PPI analysis of key drug targets. (a) PPI network of key drug targets. (b) Top 5 key drug targets under the degree algorithm. (c) Top 5 key drug targets under the bottleneck algorithm. (d) A network of “herb-active ingredient-target-disease” interactions based on hub targets.

Meanwhile, the key drug targets were subjected to PPI analysis (Figure 7(a)). In Cytoscape software, degree and bottleneck algorithm identify top 5 genes, respectively (Figures 7(b) and 7(c)). The common genes were ESR1 and PTGS2 under two algorithms. Based on the two hub targets, a network of “herb-active ingredient-target-disease” interactions was simplified (Figure 7(d)).

### 3.3. Hub Target Verification

**Figure 8:**
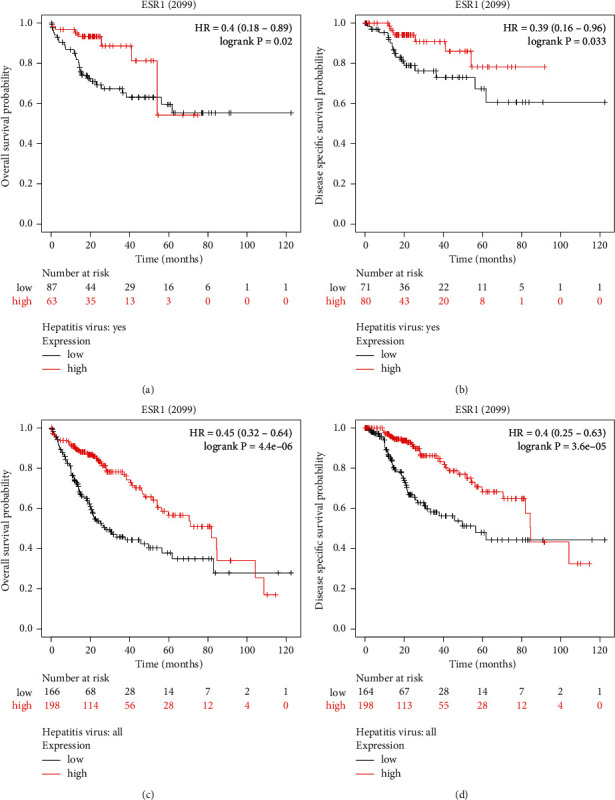
Prognostic analysis of hub targets. ESR1 expression was associated with overall survival probability (a) and disease-specific survival probability (b) in liver cancer patients with hepatitis virus. ESR1 expression was associated with overall survival probability (c) and disease-specific survival probability (d) in liver cancer patients.

The hub targets, ESR1 and PTGS2, were uploaded to the KMplotter database, and the prognostic analysis showed that ESR1 might be a tumor suppressor gene in HBV-related HCC (Figure 8).

## 4. Discussion

Globally, there are nearly 887000 deaths per year from HBV infection-related diseases, of which HBV-related HCC accounts for about 38% [32]. In China, the proportion of HCC caused by HBV is as high as 84% [32]. Research on the molecular mechanisms of HBV-related HCC development is still emerging, but there is still a lack of effective biomarkers for targeted therapy. In this study, 11 key drug targets of Curcumae Rhizoma for the treatment of HBV-related HCC were identified through a combination of data mining and network pharmacology analysis, and these key targets were characterized. This study provided new targets and ideas for the treatment of HBV-related HCC with Curcumae Rhizoma.

Through the TCMSP database and Swiss Target Prediction database, we obtained three potential active ingredients of Curcumae Rhizoma, including hederagenin, wenjine, and bisdemethoxycurcumin. Hederagenin reportedly mediated cytotoxicity to cancers via multipathways, for example, hederagenin inhibits proliferation and promotes apoptosis of cervical cancer CaSki cells by blocking the STAT3 pathway [33]. Hederagenin saponin extraction offers great potential as a antibreast cancer drug via the mitochondrial pathway [34]. By impairing autophagy, hederagenin induced ROS accumulation, potentiating the cytotoxicity of cisplatin and paclitaxel to lung cancer cells [35]. According to Liu et al., hederagenin displayed potent antihepatoma activities against human HCC HepG2 cell line [36]. Bisdemethoxycurcumin has antitumor effects exerted through a multimechanistic mode of action [37]. For example, bisdemethoxycurcumin sensitizes nonsmall cell lung cancer cells to icotinib [38]. It enhances *α*-PD-L1 antibody-mediated immune responses against bladder cancer [39]. It induces glioblastoma cell apoptosis [40]. Besides, it could cause a decrease in HCC cell viability and an increase in apoptosis [41, 42].

In terms of pathogenesis, GO-BP enrichment analysis suggested that the 11 key drug targets screened were involved in cell cycle checkpoint, DNA integrity checkpoint, and peptidyl-serine modification, all of which were associated with the development, progression, and metastasis of HCC [43–45]. The KEGG pathway enrichment results indicated that the p53 signaling pathway and arachidonic acid metabolism are enriched, which was supported by recent findings. HBV reportedly induce the abnormal lipid metabolism and activate Tregs through arachidonic acid signaling [46]. Hepatocytes with p53 inhibition escape death and senescence, becoming HCC progenitors [47]. Curcumae Rhizoma might play roles in treatment of HBV-related HCC via these biological processes and pathways.

In PPI analysis, we screened out ESR1 as a hub target. A recent study showed that ESR1 could inhibit HCC worsening [48]. ESR1 was lowly expression in liver tissues from chronic hepatitis B induced HCC in the GSE121248 dataset. Low ESR1 expression correlated with poor overall survival and disease specific survival in liver patients, no matter whether they had hepatitis virus infection. Besides, the study by Shuying Dai et al. implied that bisdemethoxycurcumin has a good affinity with ESR1 [49]. Curcumae Rhizoma, modulating ESR1, could be a potential therapeutic agent against HBV-related HCC.

Although there are some limitations in this study, such as fewer Curcumae Rhizoma active ingredients and fewer HBV-related HCC dataset, making the findings somewhat one-sided, we used bioinformatics to screen out the key drug targets of HBV-related HCC, which will lay an important foundation for subsequent research on the therapeutic targets of HBV-related HCC with Curcumae Rhizoma.

## 5. Conclusion

Based on WGCNA and network pharmacological analysis, our results illustrated that Curcumae Rhizoma might work through regulating multitargets and multipathways in HBV-related HCC. Therefore, it is suggested that we can refer to these relevant mechanisms in the future research of Curcumae Rhizoma on clinically treating HBV-related HCC. In addition, ESR1 and PTGS2 modulators are also deserved to be validated by further clinical and animal models of HBV-related HCC.

## Figures and Tables

**Table 1:** Potential active compounds of Curcumae Rhizoma.

Mol. ID	Molecule name	MW	OB (%)	DL
MOL000296	Hederagenin	414.79	36.91	0.75
MOL000906	Wenjine	282.37	47.93	0.27
MOL000940	Bisdemethoxycurcumin	308.35	77.38	0.26

**Table 2:** Potential targets of active compounds of Curcumae Rhizoma in HBV-related HCC.

Gene symbol	Protein name	logFC	Degree	Bottleneck
TOP2A	DNA topoisomerase 2-alpha	3.261963	4	1
ESR1	Estrogen receptor	−2.27525	7	9
CDK1	Cyclin-dependent kinase 1	2.029494	4	1
CYP2C19	Cytochrome P450 2C19	−1.72843	1	1
PTGS2	Prostaglandin G/H synthase 2	−1.62628	3	2
SERPINE1	Plasminogen activator inhibitor 1	−1.55996	2	1
ADH1C	Alcohol dehydrogenase 1C	−1.4645	0	0
AURKA	Aurora kinase A	1.381586	3	1
HSD17B2	17-Beta-hydroxysteroid dehydrogenase type 2	−1.15057	1	1
LYZ	Lysozyme C	1.128273	0	0
PRKDC	DNA-dependent protein kinase catalytic subunit	1.029811	3	1

**Table 3:** Enriched GO-BP and KEGG terms of potential targets.

Ontology	ID	Description	GeneRatio	BgRatio	*P* value	P adjust	*Q* value	GeneID	Count	Zscore
BP	GO:0044774	Mitotic DNA integrity checkpoint	4/11	106/18670	3.14*e* − 07	1.88*e* − 04	9.49*e* − 05	TOP2A/CDK1/AURKA/PRKDC	4	2
BP	GO:0048511	Rhythmic process	5/11	295/18670	4.07*e* − 07	1.88*e* − 04	9.49*e* − 05	TOP2A/ESR1/CDK1/SERPINE1/PRKDC	5	0.447213595
BP	GO:0031570	DNA integrity checkpoint	4/11	157/18670	1.52*e* − 06	4.10*e* − 04	2.07*e* − 04	TOP2A/CDK1/AURKA/PRKDC	4	2
BP	GO:0007093	Mitotic cell cycle checkpoint	4/11	165/18670	1.85*e* − 06	4.10*e* − 04	2.07*e* − 04	TOP2A/CDK1/AURKA/PRKDC	4	2
BP	GO:0007623	Circadian rhythm	4/11	208/18670	4.65*e* − 06	4.10*e* − 04	2.07*e* − 04	TOP2A/CDK1/SERPINE1/PRKDC	4	1
BP	GO:0000075	Cell cycle checkpoint	4/11	216/18670	5.40*e* − 06	4.10*e* − 04	2.07*e* − 04	TOP2A/CDK1/AURKA/PRKDC	4	2
BP	GO:0018105	Peptidyl-serine phosphorylation	4/11	299/18670	1.95*e* − 05	1.00*e* − 03	5.04*e* − 04	CDK1/PTGS2/AURKA/PRKDC	4	1
BP	GO:0007568	Aging	4/11	321/18670	2.57*e* − 05	0.001	5.78*e* − 04	CDK1/PTGS2/SERPINE1/PRKDC	4	0
BP	GO:0018209	Peptidyl-serine modification	4/11	322/18670	2.60*e* − 05	0.001	5.78*e* − 04	CDK1/PTGS2/AURKA/PRKDC	4	1
BP	GO:0045930	Negative regulation of mitotic cell cycle	4/11	338/18670	3.15*e* − 05	0.001	6.68*e* − 04	TOP2A/CDK1/AURKA/PRKDC	4	2
KEGG	hsa05204	Chemical carcinogenesis	3/11	82/8076	1.57*e* − 04	0.008	0.006	CYP2C19/PTGS2/ADH1C	3	−1.73205081
KEGG	hsa04913	Ovarian steroidogenesis	2/11	51/8076	0.002	0.044	0.035	PTGS2/HSD17B2	2	−1.41421356
KEGG	hsa00590	Arachidonic acid metabolism	2/11	63/8076	0.003	0.044	0.035	CYP2C19/PTGS2	2	−1.41421356
KEGG	hsa00982	Drug metabolism-cytochrome P450	2/11	71/8076	0.004	0.044	0.035	CYP2C19/ADH1C	2	−1.41421356
KEGG	hsa04115	p53 signaling pathway	2/11	73/8076	0.004	0.044	0.035	CDK1/SERPINE1	2	0

## Data Availability

The data used to support this study are available from the corresponding author upon request.
